# Role of interventional radiology in management of post-liver transplant anastomotic complications

**DOI:** 10.1186/s43055-022-00853-6

**Published:** 2022-08-02

**Authors:** Mohamed El-Gharib Abo El Maaty, Ayman Mohamed Ibrahim, Ahmed Hassan Soliman, Alaa Hamdy Mohamed

**Affiliations:** Radiodiagnosis Department, Ain Shams University, Cairo, Egypt

**Keywords:** Liver transplantation, Hepatic artery, Portal vein, Inferior vena cava, Biliary tree, Interventional radiology

## Abstract

**Background:**

Liver transplantation is considered to be the treatment of choice in cases of end-stage liver disease; however, as a major procedure, the operation is fraught with complications. The etiology, symptoms, and diagnostic methods for arterial, portal, and biliary issues are thoroughly discussed. Interventional procedures such as balloon angioplasty and stent placement in the arterial and portal systems, as well as biliary interventional procedures, are described.

**Results:**

In our study, we reviewed 25 cases of post-living donor transplanted liver, with anastomotic complications including biliary stenosis 40%, hepatic vein stenosis 20%, portal vein stenosis 16%, biliary leakage 16%, and hepatic artery stenosis or pseudo-aneurysm 16%. We had 10 cases of biliary stenosis, 7 of which were successfully treated with the insertion of an internal/external drain, and one case failed. Two patients died. We had four cases of hepatic venous obstruction with successfully implanted stents and a perfect outcome, efficacy, and patency rate of 100%. We also had two cases of hepatic artery stenosis that were perfectly managed by stent placement, with a patency rate of 100%. We came across two cases of hepatic artery pseudo-aneurysm. One case failed due to large sac size, while the other was successful. Finally, in our study, we had a 100% success rate in 5 cases of portal vein stenosis in the early postoperative period.

**Conclusions:**

Percutaneous IR was effective treatment for hepatic vein occlusion, portal vein stenosis, hepatic artery stenosis, and anastomotic biliary stricture after living donor liver transplantation. The interventional radiology team is now an integral part of the multi-disciplinary care of transplant patients. As new interventional instruments are developed and experience is gained, the outcomes of interventional treatments will continue to improve.

## Background

Liver transplantation is an established treatment for end-stage liver disease [1]. Although immunosuppression has improved post-transplant outcomes, complications such as bleeding, infections, rejection, vascular complications at the anastomotic site, and biliary complications are still possible after liver transplantation [2].

Interventional radiology procedures are preferred over similar surgical techniques because they are less invasive and show lower morbidity rates. Meanwhile, more research and expertise in the endovascular treatment of acute vascular problems in the postoperative period are required [3].

Anastomotic complications can be subdivided into vascular disorders, biliary disorders, and fluid collection [4].

Vascular complications after liver transplantation include occlusion or stenosis at the site of hepatic artery, portal, and hepatic veins anastomosis [5].

Biliary tract complications follow liver transplantation, with more recent studies indicating a 10 to 15% range. The two major types of liver transplant complications that frequently necessitate intervention are biliary obstruction and biliary leakage [6].

### Aim of the study

To highlight the role of IR in post-living donor liver transplant anastomosis complications.

## Methods

### Patients

Twenty five patients, 18 male patients (72%) and 7 female patients (28%) with a mean age ±SD = 44.2 ± 6.97 years, age range 32-58, had a living donor liver transplantation.

Before each procedure, all patients provided informed consent. Patients are drawn from the Ain Shams specialized hospital liver transplantation center and the Cairo Fatemic hospital liver transplantation unit. The study excluded patients with known coagulopathy conditions, major cardiovascular diseases, intellectual disability, or developmental disability.

### Preparation and participation

#### Full history taking

Including the date of transplantation, relation of the donor, symptoms and signs, any preoperative or postoperative complications.

#### Trans-abdominal hepatic Doppler assessment

For assessment of the resistivity index of the hepatic artery.

For assessment of the patency of portal vein, flow volume, and peak systolic velocity.

For assessment of the patency of the hepatic veins and waveform.

For assessment of the biliary radicals, perihepatic collection.

#### Laboratory tests

Liver function tests: total and direct bilirubin.

Coagulation profile: platelet count and bleeding profile.

Serum inflammatory markers: CRP and ESR.

### Magnetic resonance cholangiopancreatography

MRCP is a noninvasive technique that is effective in evaluating biliary strictures after LDLT and should be the imaging modality of choice for diagnosis in this setting. It adds the value of a detailed panoramic view of the biliary tree, allowing clinicians to identify the type, location, and severity of the biliary problem and develop an appropriate management plan.

### Multi-detector CT angiography

MDCT allows for more precise visualization of the arterial, portal, and venous phases, as well as the detection of complications. Furthermore, the ability to take thinner sections enables 3D reconstructions to create a road map plane for each transplant patient and document the changes that occurred after surgery.

### Patient preparation

Detailed explanation of the procedure.

Obtaining an informed consent.

Fasting for 6 h.

Patient was informed to stop anticoagulants.

#### Machine

The study was done at Ain Shams University Hospitals on Siemens Artis Zee and Philips monoplane machine.

#### Concerning hepatic artery stenting

- The patient lied in supine position wearing the hospital gown.
- Sterilization of the right groin was done.
- Puncture of the right femoral was done using a puncture set after local anesthesia
- 6F vascular sheath and then a 5F Cobra head catheter were introduced in right femoral artery to catheterize celiac artery.
- Diagnostic arteriography was performed from the celiac artery
- Measurements for vessel diameter and stenosis dimensions were performed after the administration of 500 units of heparin into the hepatic artery. Stenotic segments were traversed using a soft hydrophilic wire. A micro-catheter was advanced over the wire. The micro-catheter is removed, and a 6-Fr-long hydrophilic sheath will be advanced into the celiac or common hepatic artery. For stenosis > 75%, pre-stenting balloon angioplasty was performed when needed to facilitate stent catheter advancement.
- Post-stenting angioplasty up to the rated stent diameter was performed in all patients. All patients received antiplatelet medications starting on the day of the procedure.

#### For biliary system drainage

- The patient was positioned supine in the field under sterile drape
- Under ultrasound guidance, the puncture site is in the midaxillary line. A 21G needle was inserted under ultrasound guidance till reaching a branch of the common bile duct.
- A guidewire was introduced via the needle into the biliary system, common hepatic duct, till reaching the bowel.
- Once the guidewire is advanced into the bowel, the needle was removed. Multiple coaxial sheaths can then be advanced over the guidewire; the wire and the smaller, internal French sheath can then be removed, allowing a guidewire to be introduced into the larger diameter outer sheath. This combination can now be utilized to pass through strictures reaching the small intestine. If this is not achievable, external decompression with an 8F biliary drainage catheter can be utilized for a few days before attempting bowel manipulation again.

#### For portal vein angioplasty

- The operation was performed under local anesthesia.
- Under fluoroscopic and ultrasound guidance, a 22G Chiba needle was directed to the peripheral branch of the sub-capsular portal vein. The major portal vein was accessed with a guidewire. A 6–8 Fr vascular sheath was introduced after the guidewire was changed to a hydrophilic guidewire. It was possible to acquire a portal venogram. The guidewire was used to pass through the stenotic section. Angioplasty was done with the same diameter.
- Using inflating device, a careful serial elevation of the balloon pressure was achieved, and the balloon dilatation was continued until the balloon's waist was lost. The full balloon pressure was kept at that level for two minutes. The post-angioplasty portogram was acquired after the balloon dilatation was completed.

## Results

**Fig. 1:**
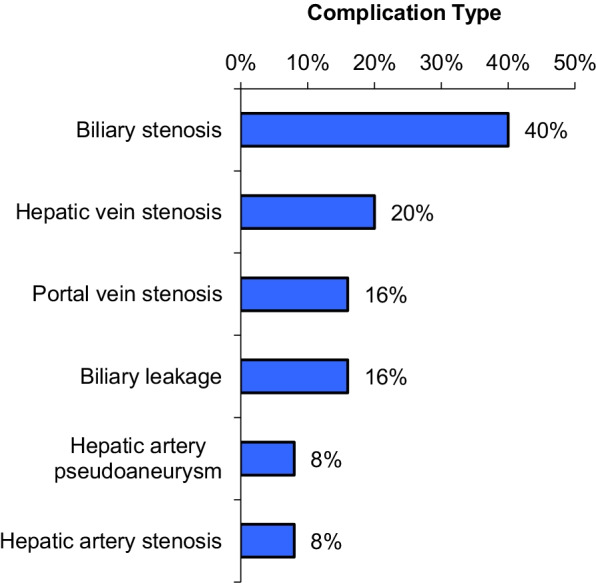
Percentage of complication type in our study cases

In our study, we reviewed 25 cases of post-living donor transplanted liver, and the anastomotic complications of the cases were as follows (Fig. 1).

**Fig. 2:**
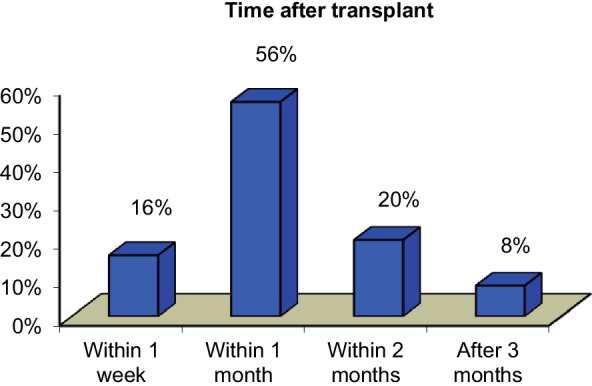
Time of appearance of complication after transplantation

For early detection of complications, cases of LDLT have regular follow-up, either laboratory investigations or US and Doppler assessment; the time interval of appearance of complications in our study was mostly within 1 month (56%) (Fig. 2).

**Fig. 3:**
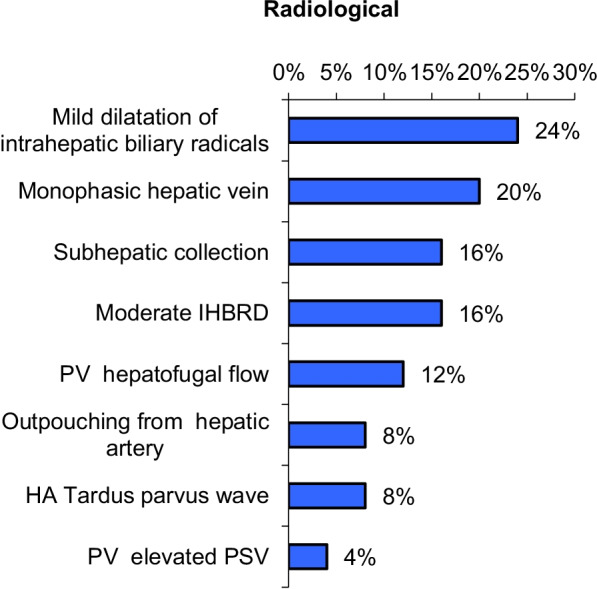
The US findings of the 25 cases

US assessment for the 25 cases is revealed in Fig. 3.

**Fig. 4:**
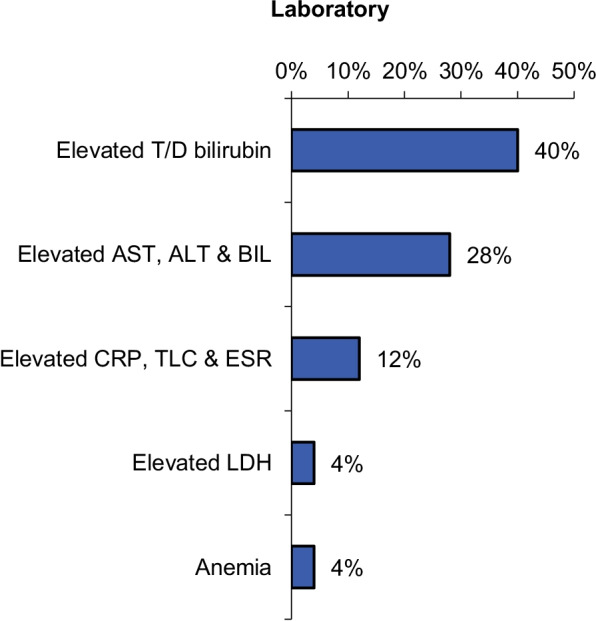
The laboratory findings percentage in our cases

Serial laboratory findings for our cases are revealed in Fig. 4.

Concerning hepatic artery stenosis, two cases of hepatic artery stenosis were successfully treated with a hepatic stent, with a success rate of 100%. Serial follow-up revealed normal resistivity index with normal laboratory results.

Technical success was defined as successful resolution of the hepatic artery stenosis with average filling of its branches with follow-up of peak systolic velocity, resistivity index of the hepatic artery with complete laboratory profile at 3 and 6 months.

In our study, we encountered two cases of hepatic artery pseudo-aneurysm; technical success was defined as complete occlusion of the pseudo-aneurysmal sac. Unfortunately, in our study, we found a failure rate of 50%.

Concerning the portal vein stenosis, we had 4 cases, and all of them showed perfect outcome with success rate of 100%.

The portal vein lumen caliber was restored, and the portal vein was properly opacified by contrast, indicating technical success, followed up by Doppler ultrasound with liver enzymes and LDH assessment.

Unfortunately, one case died out of COVID infection after 1 month and loss of serial follow-up.

Concerning cases of hepatic vein stenosis, we had 4 cases, with stent insertion that showed success rate of 100%.

Technical success was defined as restoration of the hepatic vein lumen caliber with resolution of the collaterals.

Concerning the biliary system, we had 10 cases of biliary stenosis.

Technical success is defined by cholangiogram with resolution of intrahepatic dilatation of the biliary radicals by follow-up ultrasound and normalization of the total, direct bilirubin and alkaline phosphatase.

Seven of them were treated with an internal/external drain or a plastic stent. Because of the tight distal stenosis, one case was referred for ERCP. Two cases died as a result of COVID, while the third died as a result of respiratory failure.

We had four cases of biliary leakage, all of which required Pigtail insertion under complete US guidance, with a success rate of 100% for perfectly seated pigtails. Two cases demonstrated resolution of the sub-hepatic collection, while the other two demonstrated organization of the sub-hepatic collection.

**Fig. 5:**
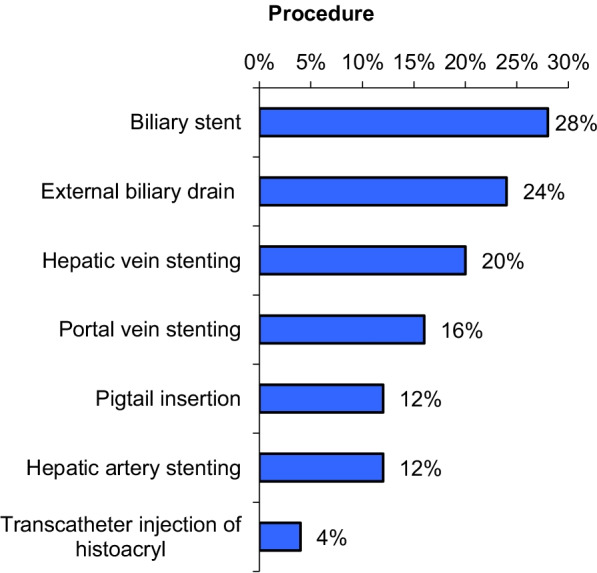
The percentage of procedures done in our study

In this figure, we summarize the procedures done in our study (Fig. 5).

**Fig. 6:**
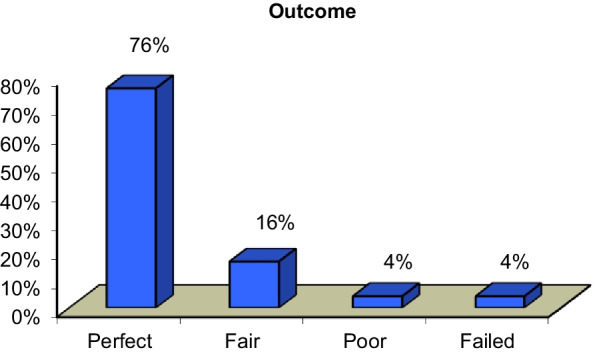
The percentage of procedures early outcome of our cases

The cases' outcomes were classified based on the previously defined technical success, with a complete success rate of 76% (19 cases). In approximately 16% of cases, a favorable outcome was obtained (4 cases). In one case, a pseudo-aneurysm with residual flow in the pseudo-aneurysmal sac, the outcome was poor. One case was unsuccessful. Other cases of large hepatic artery pseudo-aneurysm were referred for surgical intervention (Fig. 6).

**Fig. 7:**
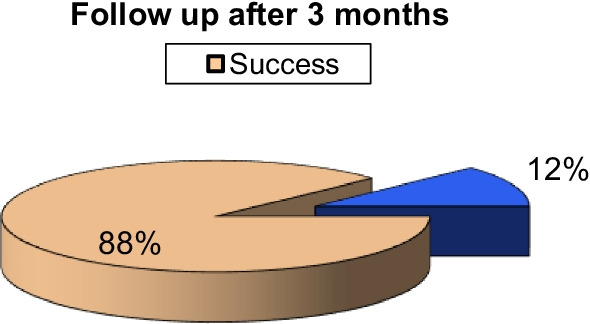
The percentage of procedures follow-up result after 3 months done in our study

Follow-up after 3 months of the procedure revealed an increased percentage of success rate of 88%, raised from 19 to 22 case, as two cases of biliary leakage revealed resolution of any residual collection after Pigtail insertion and one case of pseudo-aneurysm revealed complete thrombosis of the sac. One case died as a result of COVID disease, while another with biliary stenosis showed mild improvement after external draining and was referred for ERCP (Fig. 7).

In this table, we compare anastomotic complication with early outcome; a significant relationship was found in cases of hepatic artery pseudo-aneurysm, but the obtained outcome was not perfect. However, more cases needed to be enrolled in order to reach a reliable conclusion in this matter (Table 1).

**Table 1:** The relation between the complication, time after transplant, and the early outcome

	Outcome		Test value	*P* value	Sig.
	Perfect	Not perfect			
	No. = 18	No. = 7			
Complications					
Portal vein stenosis	3 (16.7%)	1 (14.3%)	0.021*	0.884	NS
Hepatic artery pseudo-aneurysm	0 (0.0%)	2 (28.6%)	5.590*	0.018	S
Biliary stenosis	7 (38.9%)	3 (42.9%)	0.033*	0.856	NS
Biliary leakage	2 (11.1%)	2 (28.6%)	1.143*	0.285	NS
Hepatic vein stenosis	4 (22.2%)	1 (14.3%)	0.198*	0.656	NS
Hepatic artery stenosis	2 (11.1%)	0 (0.0%)	0.845*	0.358	NS
Time after transplant					
Within 1 week	2 (11.1%)	2 (28.6%)	1.899*	0.594	NS
Within 1 month	10 (55.6%)	4 (57.1%)			
Within 2 months	4 (22.2%)	1 (14.3%)			
Within 3 months	2 (11.1%)	0 (0.0%)			

*: Chi-square test

Concerning the time after transplant, no significant relationship was found between the early outcome and the time after transplant. This can be attributed to the regular follow-up in our institute, which ensures the early detection of complications, and the multi-disciplinary management, which necessitates treatment as soon as possible (Table 1).

In this table, we compare the anastomotic complications that occurred with the 3-month follow-up, and we found that we had a high technical success rate with a low failure rate. As a result, no significant relationship could be found between a specific procedure and an abnormal outcome.

Also, because of the regular follow-up, the time after transplant was found to have no significant relationship with either the early outcome or the 3-month follow-up (Table 2).

**Table 2:** The relation between the complication, time after transplant, and the outcome after 3-month follow-up

	Follow-up after 3 months		Test value	*P* value	Sig.
	Success	Failed			
	No. = 22	No. = 3			
Complications					
Portal vein stenosis	3 (13.6%)	1 (33.3%)	0.762*	0.383	NS
Hepatic artery pseudo-aneurysm	1 (4.5%)	1 (33.3%)	2.973*	0.085	NS
Biliary stenosis	9 (40.9%)	1 (33.3%)	0.063*	0.802	NS
Biliary leakage	4 (18.2%)	0 (0.0%)	0.649*	0.420	NS
Hepatic vein stenosis	5 (22.7%)	0 (0.0%)	0.852*	0.356	NS
Hepatic artery stenosis	2 (9.1%)	0 (0.0%)	0.296*	0.586	NS
Time after transplant					
Within 1 week	3 (13.6%)	1 (33.3%)	1.664*	0.645	NS
Within 1 month	12 (54.5%)	2 (66.7%)			
Within 2 months	5 (22.7%)	0 (0.0%)			
Within 3 months	2 (9.1%)	0 (0.0%)			

*P* value > 0.05: non-significant; *P* value < 0.05: significant; *P* value < 0.01: highly significant

*: Chi-square test

### Illustrative cases

#### Case no. 1

**Fig. 8:**
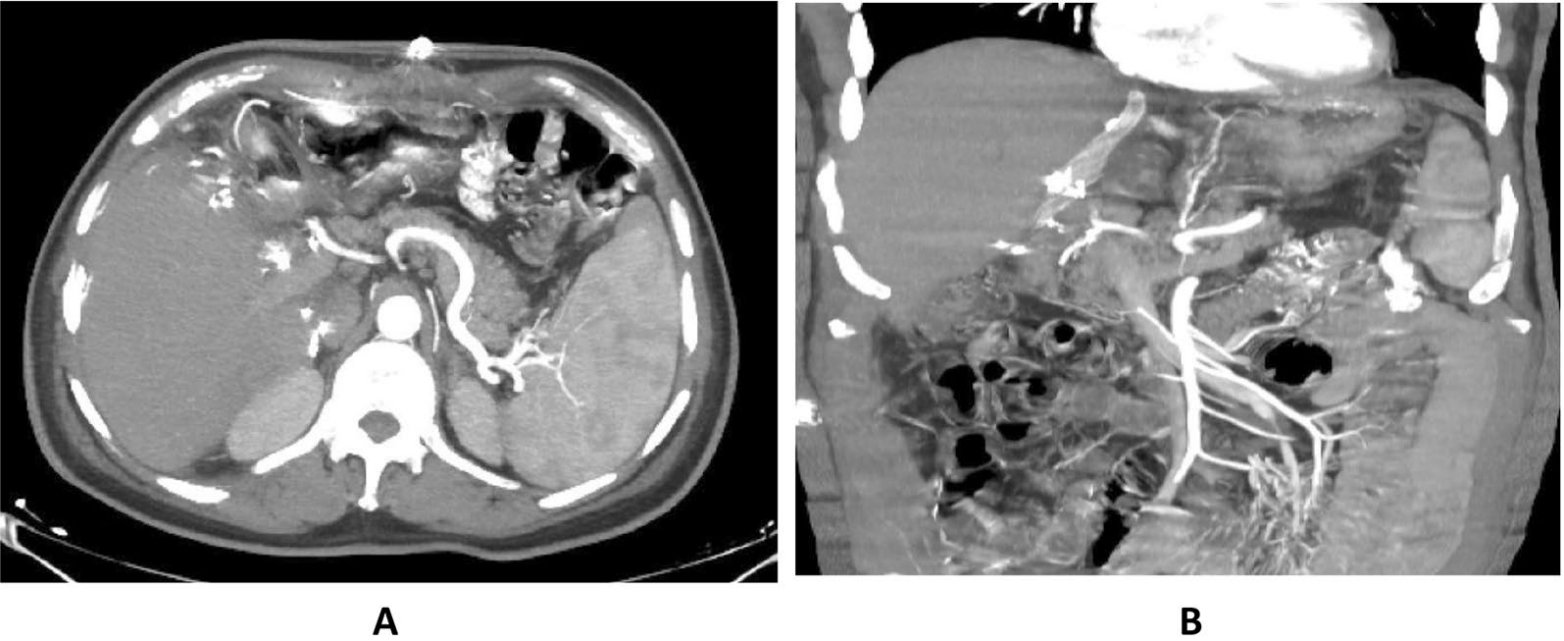
**A** Axial contrast-enhanced CT images (Mini MIP) showing markedly attenuated caliber of the hepatic artery. **B** Coronal CT images (Mini MIP); the hepatic artery cannot be traced at the porta hepatis

**Fig. 9:**
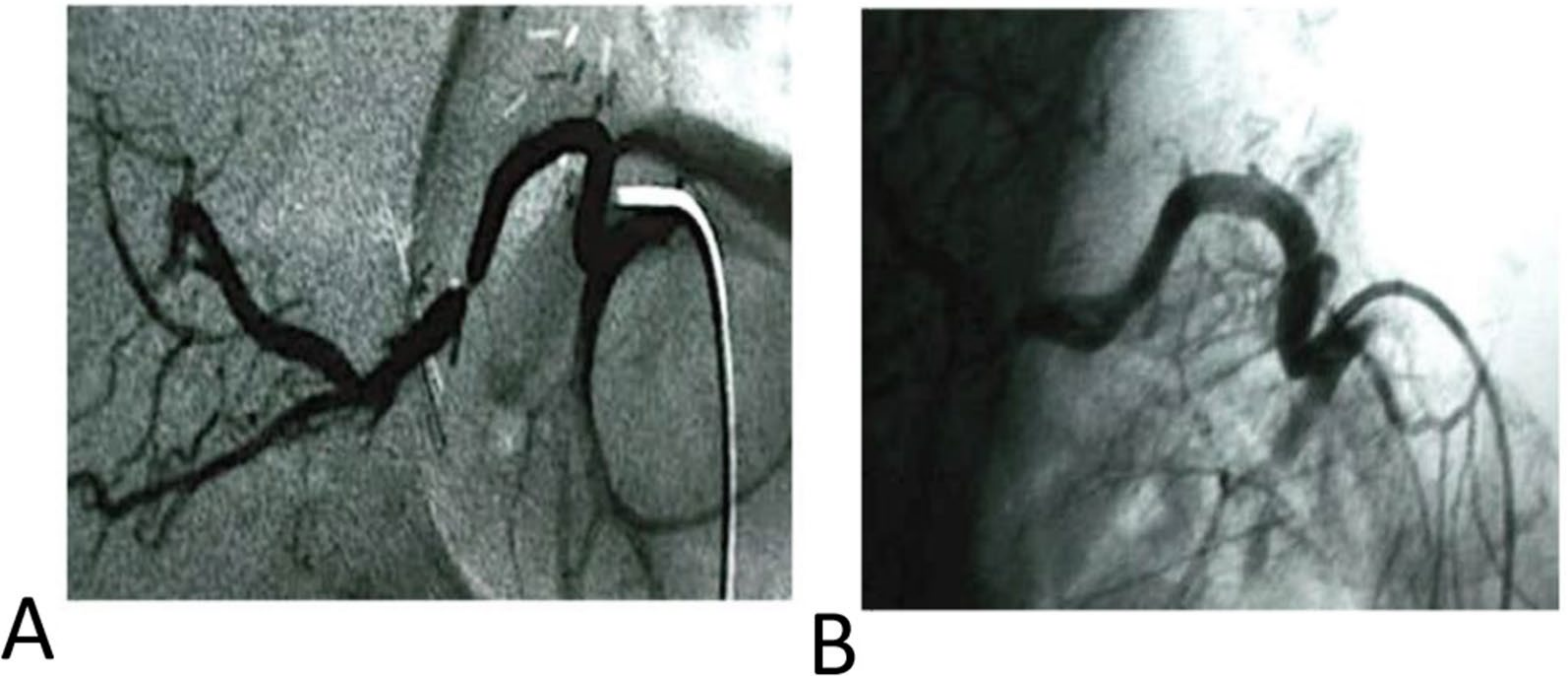
**A** Diagnostic angiography of the hepatic artery revealed segmental significant stenosis. **B** Diagnostic angiography after stent placement

A male patient, 58 years old, presented by elevated RI of the hepatic artery exceeding 0.8 one week after the procedure with progressive elevation of the PSV. The decision was hepatic artery stenting with perfect outcome. Follow-up after one, 3 months revealed normal RI and PSV (Figs. 8, 9).

#### Case no. 2

**Fig. 10:**
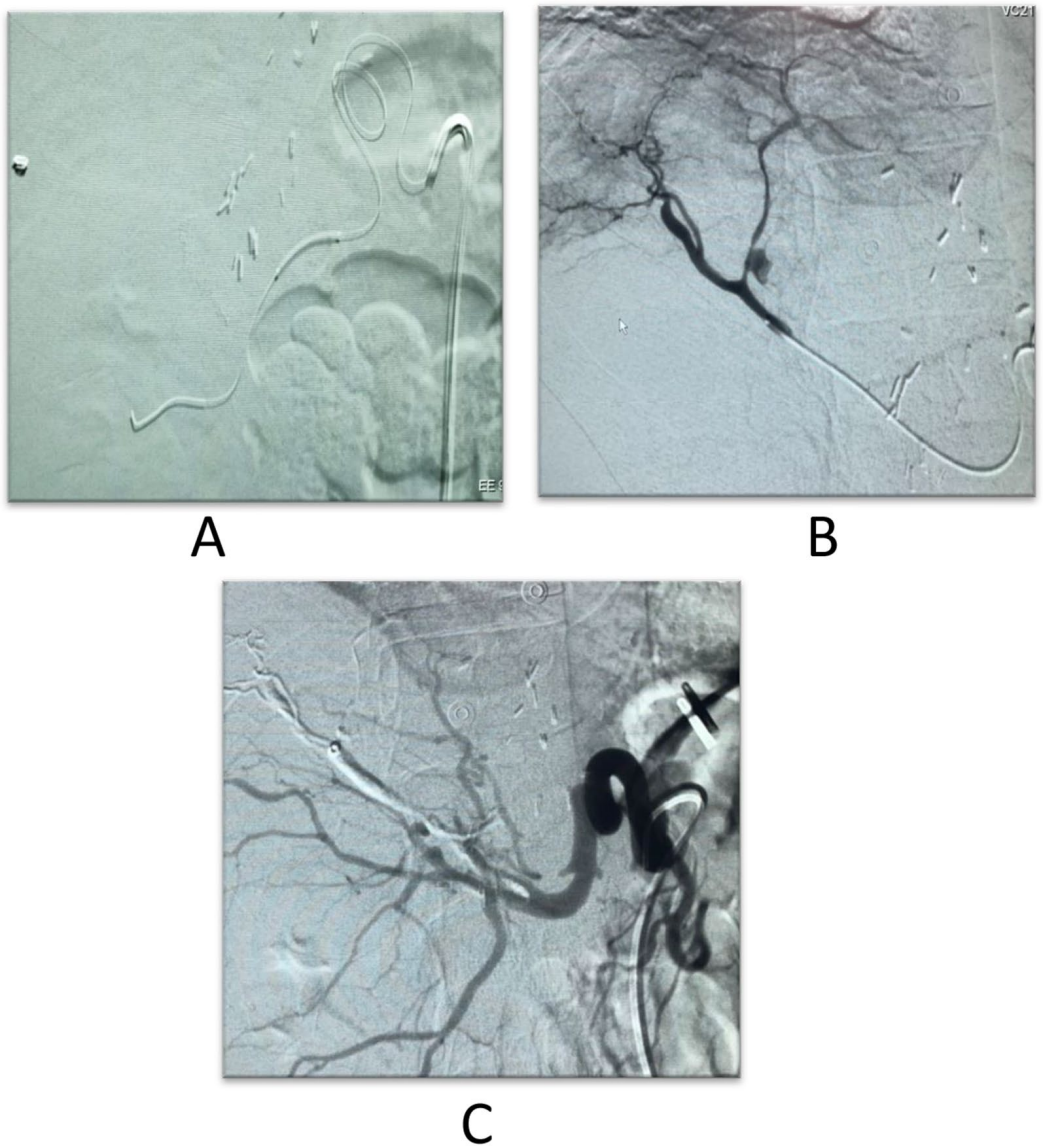
**A** The micro-catheter in the hepatic artery. **B** A pseudo-aneurysmal sac arising from the anterior branch of the hepatic artery. **C** Non-opacification of the pseudo-aneurysmal sac after histoacryl injection

A 38-year-old male patient with a 1-week follow-up revealed out-pouching from the right anterior branch with Yin Yang Sing, Normal Labs, diagnostic angiography revealed pseudo-aneurysmal dilatation from the right hepatic artery anterior branch, trans-catheter injection of histoacryl was performed, and the pseudo-aneurysmal sac was completely occluded (Fig. 10).

Follow-up US revealed near total occlusion of the sac; however, with residual flow detected within the sac, follow-up US after month revealed total occlusion of the sac with no flow detected.

#### Case no. 3

**Fig. 11:**
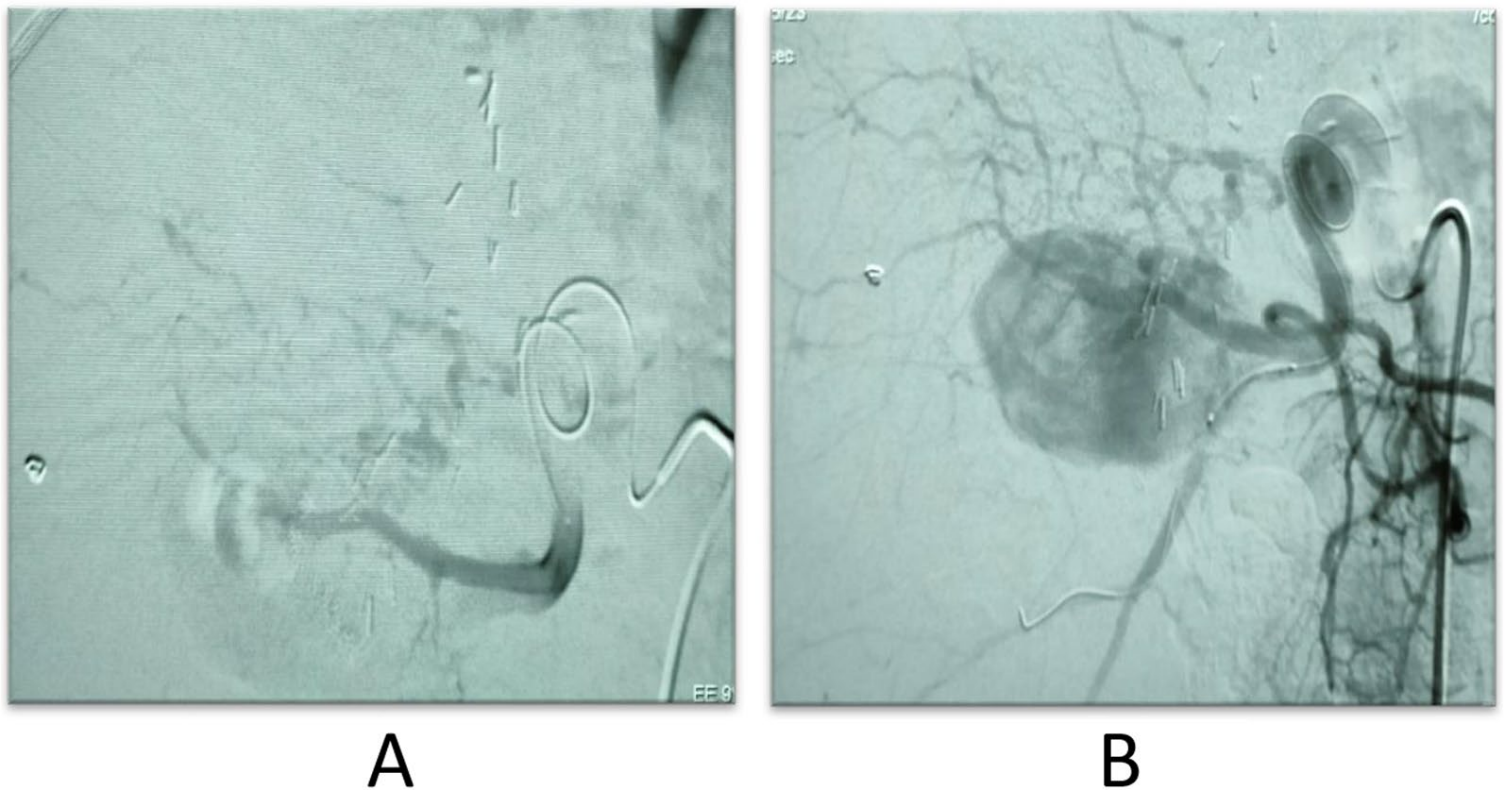
**A** The micro-catheter in the hepatic artery. **B** The large pseudo-aneurysmal sac arising from the hepatic artery

A 41-year-old female patient had a much larger out-pouching from the anterior branch of the right hepatic artery, which showed turbulent internal flow on Doppler interrogation, and the pseudo-aneurysm size was large, with failed coil insertion due to the large neck size. It was referred for surgical intervention as well (Fig. 11).

#### Case no. 4

A 56-year-old male patient, routine laps after 3 months, revealed elevated LDH, Doppler examination revealed portal vein hepatofugal flow, and CT angiography revealed significant luminal stenosis at the site of anastomosis. Diagnostic angiography confirmed the portal vein stenosis, and portal vein stenting was performed with no complications.

**Fig. 12:**
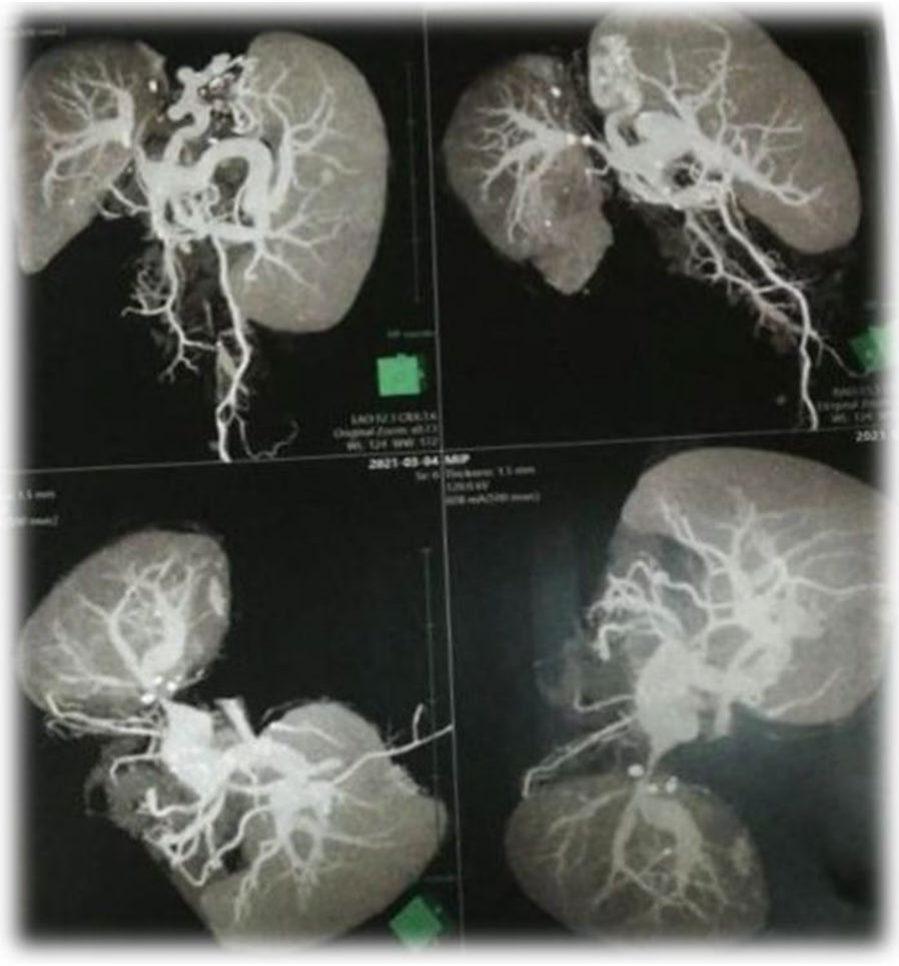
CT angiography reconstruction images showed significant luminal stenosis at site of anastomosis of the portal vein

**Fig. 13:**
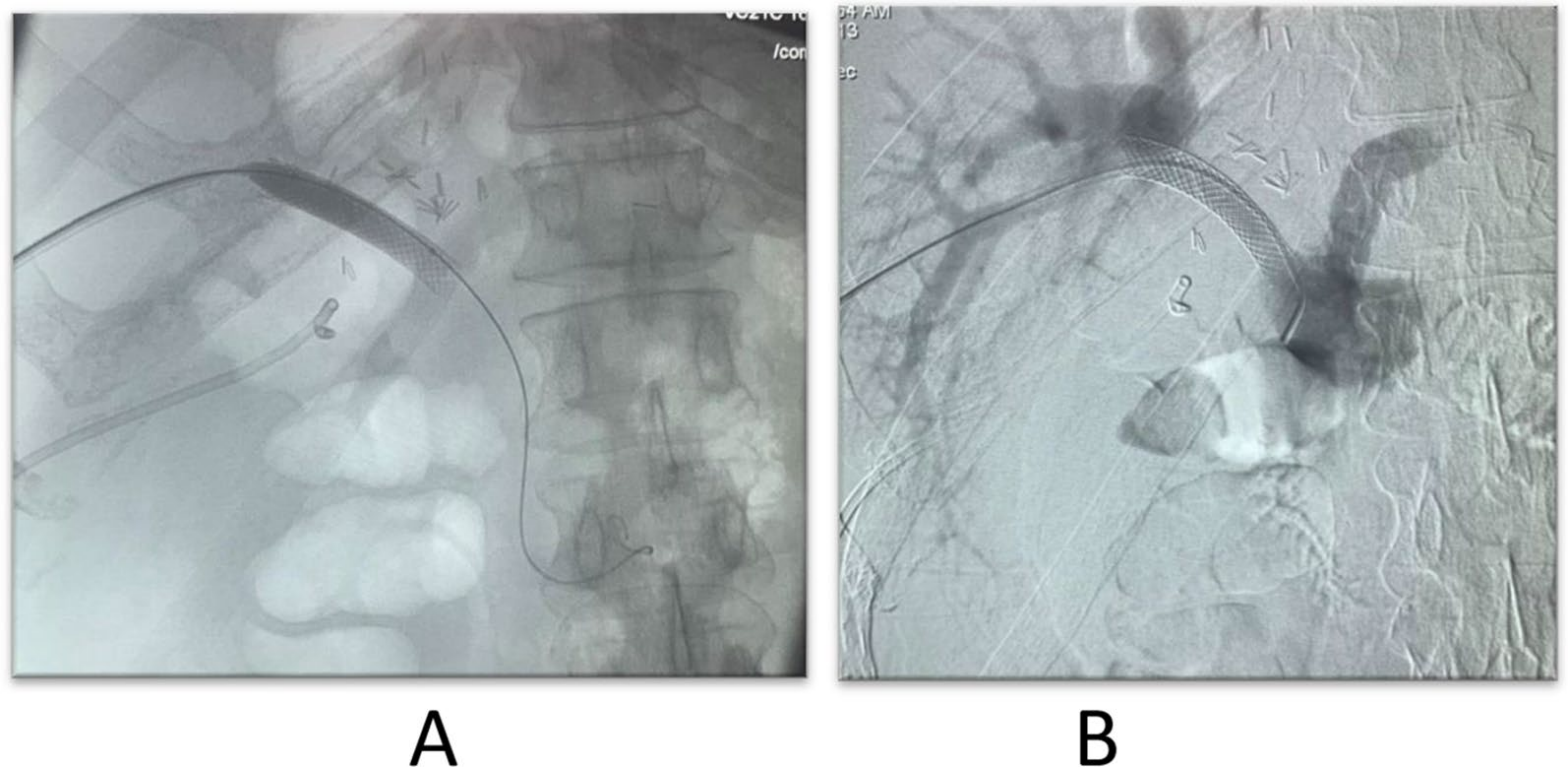
**A** A hydrophilic wire introduction within the portal vein with well-placed stent. **B** A well-placed portal vein stent after contrast injection

After 1 month follow-up revealed normalization of LDH and normal portal venous flow (Figs. 12, 13).

#### Case no. 5

**Fig. 14:**
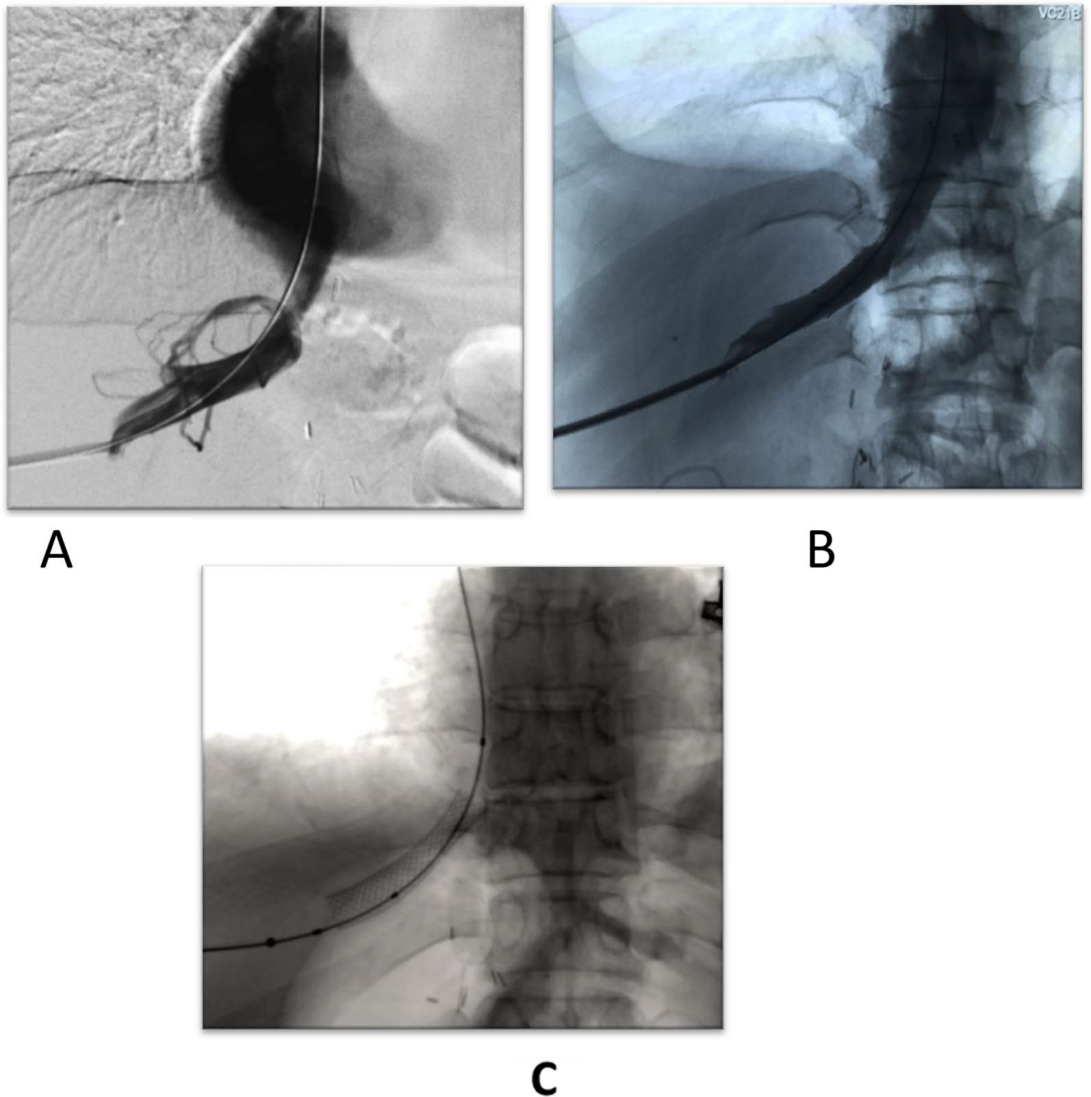
**A** Multiple collaterals related to the hepatic vein. **B** Proper contrast opacification within stent with resolution of collaterals. **C** A well-placed stent within hepatic vein

A male patient, 50 years old, had monophasic venous flow of the hepatic vein and balloon angioplasty, and hepatic vein stenting was done successfully with no complications encountered (Fig. 14).

#### Case no. 6

**Fig. 15:**
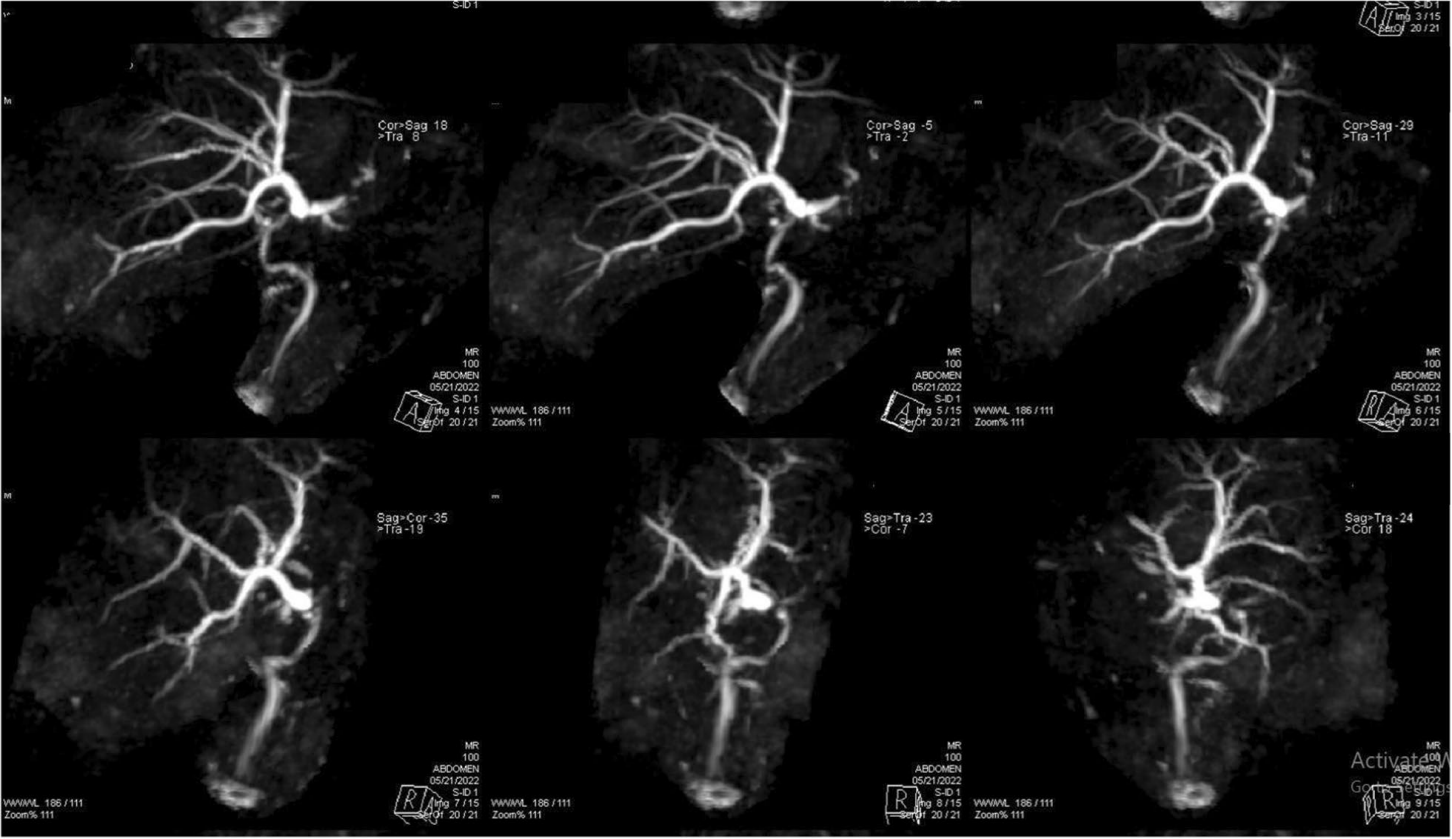
MRCP images showing mild intrahepatic biliary channels dilatation with focal stricture at the site of anastomosis

**Fig. 16:**
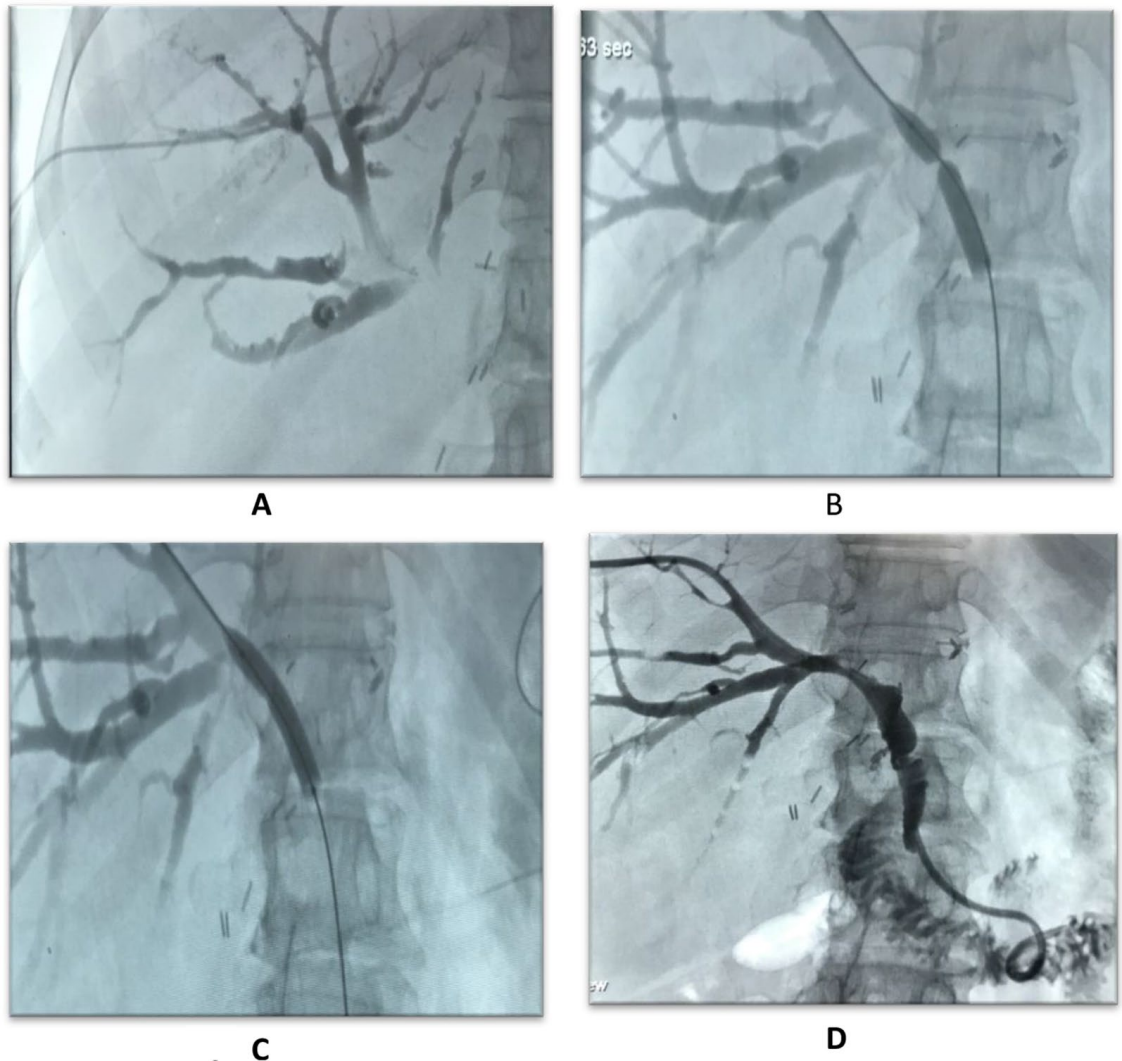
**A** A transhepatic insertion of an angiocath with contrast opacification of the biliary radicals. **B** A hydrophilic wire inserted in the CBD with wasting of the balloon, **C** The resolved wasting on balloon inflation. **D** A well-placed internal/external drain with opacification of the bowel loops denoting proper drainage

A 39-year-old male patient presented by elevated total/direct bilirubin, with US showing moderate dilatation of the intrahepatic biliary radicals, diagnosed as biliary stricture (Figs. 15, 16).

#### Case no. 7

**Fig. 17:**
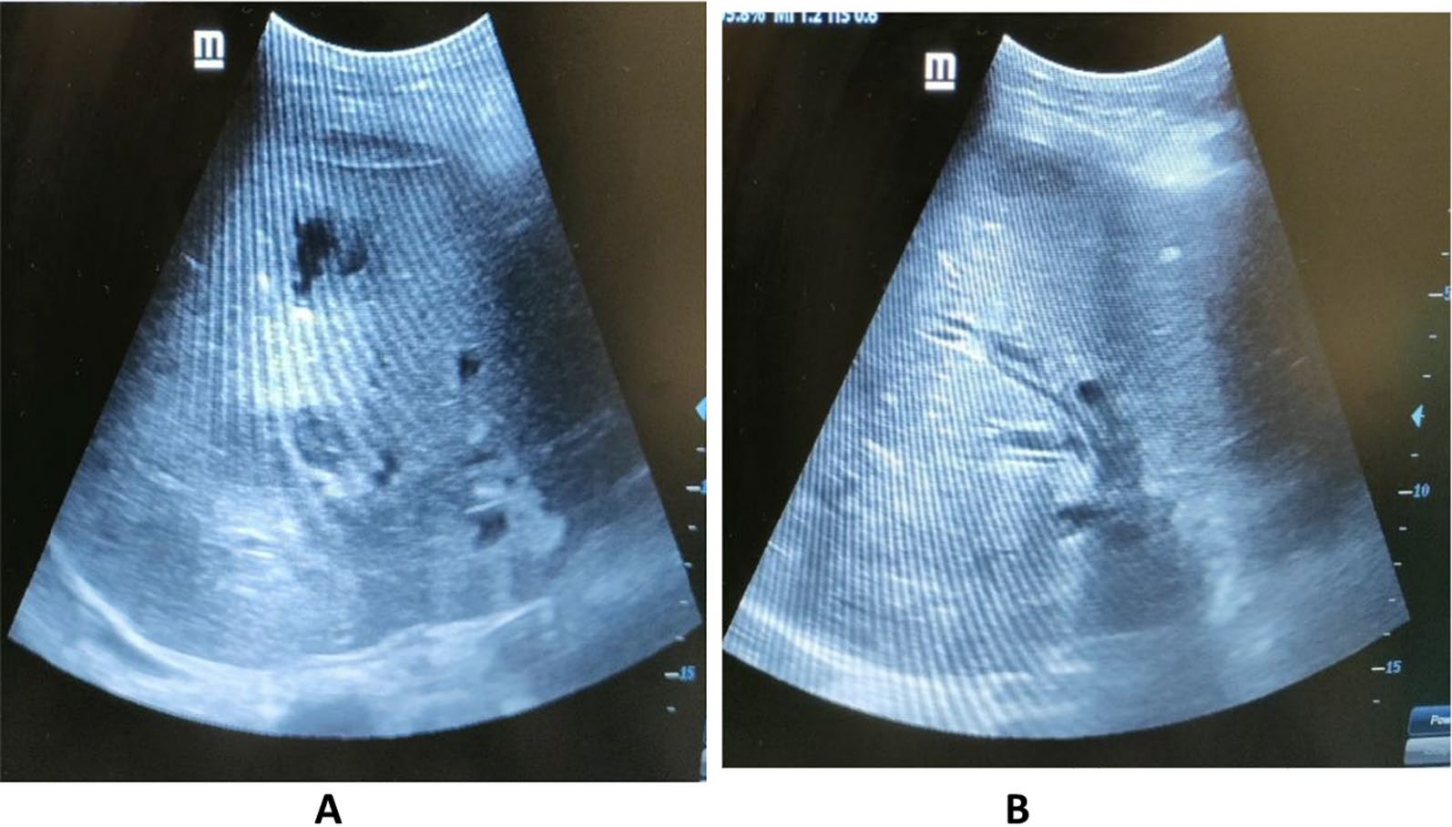
**A** A US examination revealed localized intrahepatic collection. **B** Mild dilatation of intrahepatic biliary radicals

A 39-year-old male patient presented by elevated total and direct bilirubin, with US showing mild dilatation of intrahepatic biliary radicals with two small cholangectatic abscesses (Fig. 17).

**Fig. 18:**
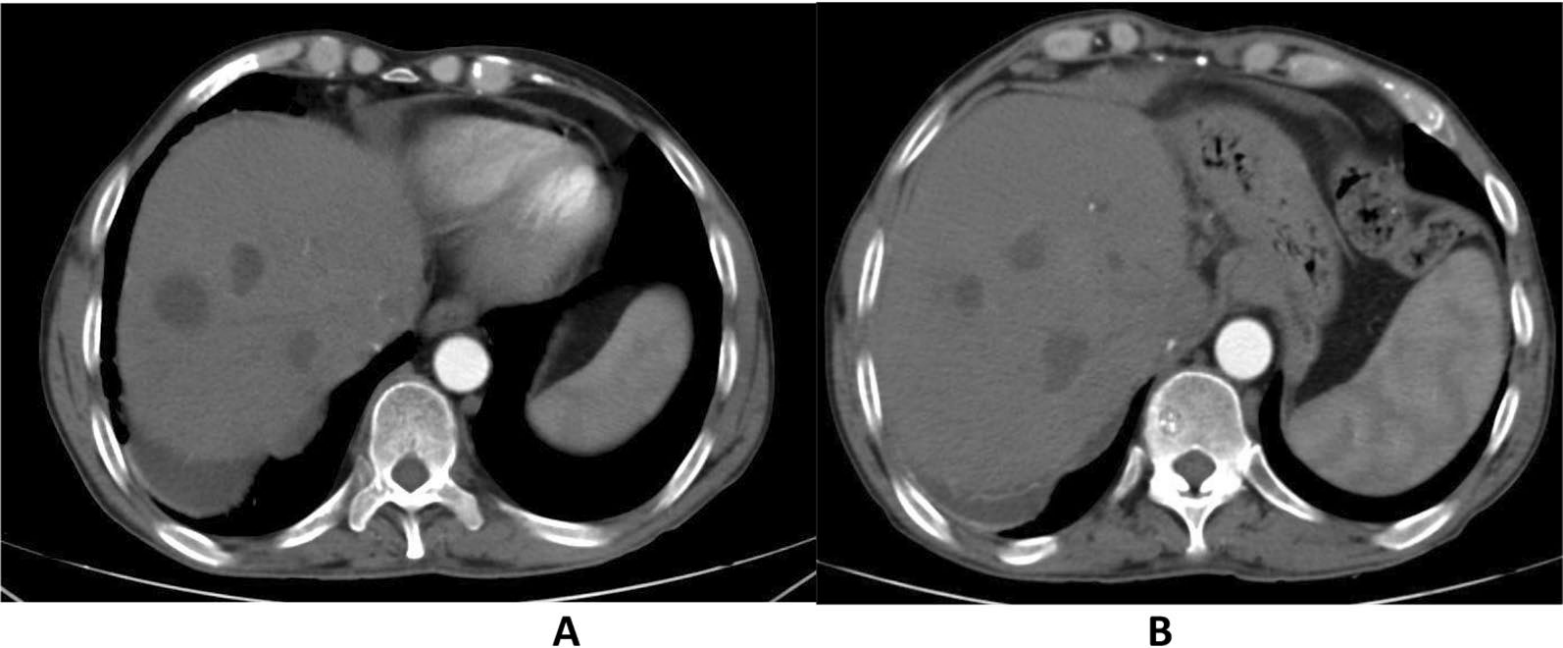
Contrast enhanced CT, arterial phase showing multiple hypodense cystic lesions with marginal enhancement denoting abscesses formation. (**A**) showing cholangectatic abscesses in segment VII, (**B**) showing cholangectatic abscesses in segments V & VI

**Fig. 19:**
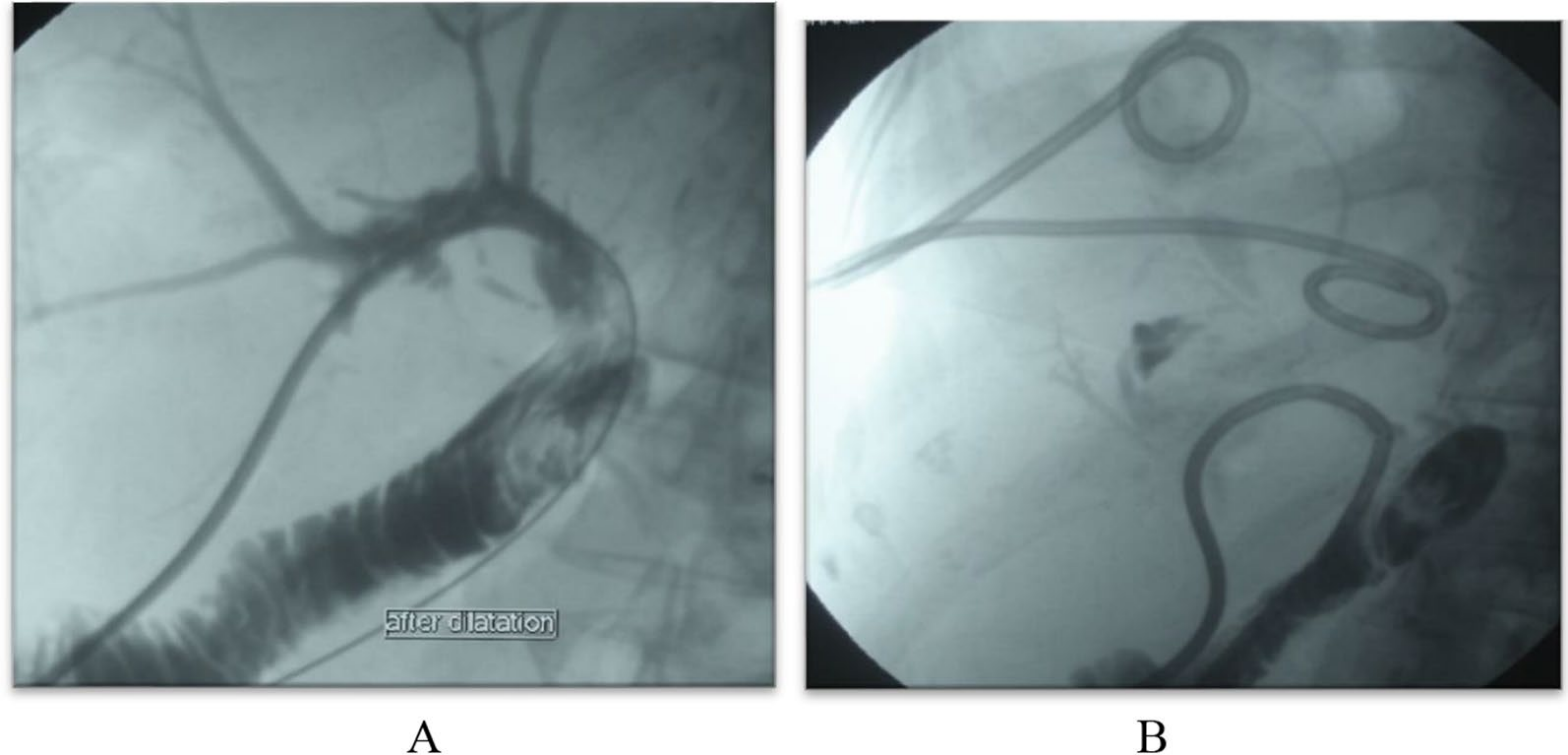
A well-placed internal/external drain with opacification of the bowel loops denoting proper drainage (**A**), with two drainage pigtails for the abscesses (**B**)

Serial follow-ups revealed that progression of size of the cholangectatic abscesses as seen in the attached contrast-enhanced CT images with follow-up laboratories showed elevated total and direct bilirubin and thus the decision was agreed for percutaneous transhepatic drainage of the biliary system along with pigtails insertion in the abscesses for drainage and analysis as well (Figs. 18, 19).

## Discussion

We wanted to emphasize the importance of interventional radiology in the multi-disciplinary management of liver transplantation in our study, which reviewed 25 cases of liver donor transplantation at our institution. We look at how interventional radiology procedures can be used to treat the resulting post-anastomotic problems.

Technical factors such as a tight suture line, a difference in portal vein size, portal vein tension or torsion, or the use of a bypass graft can all contribute to early portal vein stenosis as a result of portal vein anastomosis [7]. Later on, intimal hyperplasia or fibrosis surrounding the anastomosis can cause portal vein stenosis [8].

In our study, we had a 100% success rate in 5 cases of portal vein stenosis in the early postoperative period. Shibata et al. performed IR on 43 patients with portal vein stenosis after LDLT, with follow-up ranging from 5 to 169 months (mean, 119 months). Technical success was obtained in 65 of 66 sessions (98%) and 42 of 43 patients (98%) [9].

Chick et al. reported a 98% technical success rate and an 88% 2-year primary patency rate in 36 patients [10]. According to Denys et al.’s long-term patency study, stenosis recurred after a mean follow-up of 6.3 months in 50% of balloon venoplasty patients. Stent patency was excellent, with a 100% patency rate after a mean follow-up of 47 months [11]. In the serial follow-up, these results are seen to match our results of efficacy of portal vein stenting with proper patency. In the management of portal vein stenosis after liver transplantation, we can consider portal vein stenting with or without balloon angioplasty as the first option [12].

Hepatic artery stenosis is a potentially fatal condition that develops soon after receiving a liver transplant. Patients who develop this complication are more likely to experience graft rejection or biliary issues. Hepatic artery stenosis at the anastomosis can be caused by the operating approach, acute cellular rejection, or intimal dissection [13]. Hepatic artery stenosis is common at anastomoses and may be caused by the operative technique, acute cellular rejection, or intimal dissection.

The most successful treatment is surgical revascularization or re-transplantation; however, repeated operations can be difficult due to extensive fibrosis or inflammation surrounding the hepatic artery or a lack of an adequate artery for reconstruction [8]. As a result, endovascular procedures such as balloon angioplasty and stent application have evolved as less invasive alternatives to surgical procedures [14].

We had two cases of hepatic artery stenosis in our study that were perfectly managed by stent placement, with a perfect outcome and patency rate of 100% in the follow-up period. The number of cases of hepatic artery stenosis is decreasing due to increased surgeon experience and the evolution of many high-quality devices that facilitate this such challenging anastomosis, and thus, hepatic artery stenosis is considered one of the least anastomotic complications to occur.

Le et al. reported a technical success rate of 95% (59 of 62) of cases [15], while Vignali et al. reported a technical success rate of 75% (7 cases out of 8 cases), with one case complicated by intimal dissection during the procedure necessitating surgical intervention [16]. Hamby et al. reviewed 23 cases of hepatic artery interventions and found a 97% technical success rate [17]. Cotroneo et al. achieved a technical success rate of 100% for four cases of hepatic artery stenosis, with a patency rate of 100% for 18–25 months of follow-up [18]. Hepatic artery pseudo-aneurysm is a rare complication of liver transplantation that usually occurs in the first month after the procedure, and they occurred either iatrogenic or less likely associated with localized collection [19].

We encountered two cases of hepatic artery pseudo-aneurysm in our study. One case failed due to large sac size, while the other had a good outcome with residual flow after intervention; however, the sac was completely thrombosed 1 month after transplantation.

Hepatic vein stenosis is common in LDLT. It has been linked to differences in hepatic vein size, a tight suture line, twisting or kinking, and numerous anastomoses in the early post-transplant period. Late-onset stenosis is typically caused by peril-anastomotic fibrosis, but it can also be caused by edema-induced compression or liver graft regeneration [8].

In our study, we had four cases of hepatic venous obstruction that were successfully treated with stents and had a perfect outcome, efficacy, and patency rate of 100%.

Toshiya et al. performed IR on 48 patients with hepatic vein occlusion following LDLT, with follow-up ranging from 1 to 182 months (median, 51.5 months). Technical success was achieved in 92 of 93 sessions (99%) and in 47 of 48 patients (98%) [12].

Anastomotic biliary stricture is the most common biliary complication. Some studies have suggested that biliary stricture occurs more frequently in post-LDLT patients due to the narrow width of the anastomotic part of the bile duct, anatomical diversity of the bile ducts, or the difficult nature of the surgical operation [20].

ERCP is typically performed on transplant recipients in order to diagnose and treat strictures and leaks. ERCP is frequently effective for choledochocholedochostomy biliary reconstructions, but for many transplant recipients, a Roux-en-Y hepaticojejunostomy is too difficult or time-consuming for the endoscopic method. Percutaneous transhepatic cholangiography and percutaneous transhepatic biliary drainage are reserved for cases in which ERCP fails or is intractable due to clinical or anatomical reasons [21].

In our study, we had 10 cases of biliary stenosis, 7 of which were successfully treated with the insertion of an internal/external drain, while one case failed and was referred for ERCP. Two cases died as a result of COVID and respiratory failure, with no serial follow-up. Our technological success rate was 90%.

Toshiya et al. performed IR on 52 patients with anastomotic biliary stenosis following LDLT, with follow-up ranging from 5 to 206 months (median, 100 months). In 43 of 52 patients, clinical success was observed (83%) [12].

Enrico et al. achieved resolution of the biliary complication with a good clinical course was achieved in 13 of the 17 cases treated (76.5%), 5 of 8 cases were treated with percutaneous transhepatic biliary drainage, and 8 of 9 cases were treated with percutaneous transhepatic biliary drainage combined with stricture dilation with a balloon catheter for fibrotic post-transplantation strictures. There were no major complications associated with transhepatic biliary drainage; however, there were two cases of mild hemobilia caused by iatrogenic communication of the bile duct with a branch of the suprahepatic veins, which resolved spontaneously after a few days [22].

## Limitations

Our study had several limitations, including a small sample size and a short time interval for follow-up. Unfortunately, the study period coincided with the spread of COVID-19, which limited transplantation procedures due to the fear of a fulminant disease process during the immunosuppressive state of the post-transplant.

## Conclusions

Living donor liver transplantation is a procedure with many related complications, and recurrences are still a concern, and the interventional radiology team is now a corner stone of the multi-disciplinary management. Percutaneous IR is a minimally invasive, safe, and effective treatment for hepatic vein occlusion, portal vein stenosis, hepatic artery stenosis, and anastomotic biliary stricture following LDLT. As new interventional instruments are developed and experience is gained, the outcomes of interventional treatments will continue to improve.

## Data Availability

The data and material used in this study are available.

## Abbreviations

IR: Interventional radiology
CRP: C-reactive protein
ESR: Erythrocyte sedimentation rate
LDLT: Living donor liver transplantation
US: Ultrasound
LDH: Lactate dehydrogenase
ERCP: Endoscopic retrograde cholangiopancreatography
